# Combining chromosomal microarray and clinical exome sequencing for genetic diagnosis of intellectual disability

**DOI:** 10.1038/s41598-023-50285-z

**Published:** 2023-12-20

**Authors:** Jaewon Kim, Jaewoong Lee, Dae-Hyun Jang

**Affiliations:** 1 Department of Physical Medicine and Rehabilitation, Incheon St. Mary’s Hospital, College of Medicine, The Catholic University of Korea, Seoul, Republic of Korea; 2 Department of Laboratory Medicine, Incheon St. Mary’s Hospital, College of Medicine, The Catholic University of Korea, Seoul, Republic of Korea; 3 Medical Genetics and Rare Disease Center, Incheon St. Mary’s Hospital, College of Medicine, The Catholic University of Korea, Seoul, Republic of Korea

**Keywords:** Neurodevelopmental disorders, Genetic testing

## Abstract

Despite the current widespread use of chromosomal microarray analysis (CMA) and exome/genome sequencing for the genetic diagnosis of unexplained intellectual disability (ID) in children, gaining improved diagnostic yields and defined guidelines remains a significant challenge. This is a cohort study of children with unexplained ID. We analyzed the diagnostic yield and its correlation to clinical phenotypes in children with ID who underwent concurrent CMA and clinical exome sequencing (CES). A total of 154 children were included (110 [71.4%] male; mean [SD] age, 51.9 [23.1] months). The overall diagnosis yield was 26.0–33.8%, with CMA contributing 12.3–14.3% and CES contributing 13.6–19.4%, showing no significant difference. The diagnostic rate was significantly higher when gross motor delay (odds ratio, 6.69; 95% CI, 3.20–14.00; *P* < 0.001), facial dysmorphism (odds ratio, 9.34; 95% CI 4.29–20.30; *P* < 0.001), congenital structural anomaly (odds ratio 3.62; 95% CI 1.63–8.04; *P* = 0.001), and microcephaly or macrocephaly (odds ratio 4.87; 95% CI 2.05–11.60;* P* < 0.001) were presented. Patients with only ID without any other concomitant phenotype (63/154, 40.9%) exhibited a 6.3–11.1% diagnostic rate.

## Introduction

Intellectual disability (ID) refers to a neurodevelopmental disorder characterized by limitations in both intellectual and adaptive functioning, typically with an intelligence quotient (IQ) score of at most 70 or at least two standard deviations below the population mean. The estimated global incidence of ID in children is 1–3%^1–3^. For children aged 5 years and younger, ID is diagnosed as a global developmental delay characterized by a significant delay in two or more of the following developmental domains: cognitive, speech, social/personal, gross/fine motor, and activities of daily living. The global developmental delay is considered a predictor of future ID diagnosis^4,5^. Notably, genetic diagnosis of ID is complicated because children with this disease show clinical diversity and genetic heterogeneity^6^. Despite being a costly, time-consuming, and complicated process, genetic diagnosis holds potential as it enables parents to complete diagnostic odyssey and obtain detailed information while facilitating family planning decisions.

Single nucleotide variants (SNVs), small insertions and deletions (indels), and structural variations (SVs), specifically copy number variations (CNVs), have been recognized as significant genetic causes of ID. Children with ID were evaluated for CNV using chromosomal microarray analysis (CMA) and sequencing-based assays. The 2021 American College of Medical Genetics and Genomics (ACMG) guidelines recommend exome/genome sequencing (ES/GS) as a first- or second-tier test. However, given the high cost of ES/GS, the guideline suggests performing CMA or targeted gene sequencing first and then ES/GS, if necessary^7,8^. Notably, research has revealed that while GS offers the highest diagnostic yield. However, considering cost-effectiveness and a respectable detection rate, ES is also recommended as a primary testing for the detection of genetic diseases^7,9^. Although the diagnostic yield of targeted gene sequencing is not below that of CMA, studies objectively comparing the diagnostic yields of CMA and ES for ID cases remain lacking^6,10–12^. In previous studies, negative CMA results have been associated with a high rate of loss to follow-up or undiagnosed without further genetic testing, and the final diagnosis rate would have increased if targeted gene sequencing had been performed^13,14^. Therefore, this study investigated the diagnostic yields of concurrent CMA and clinical ES (CES) testing in order to identify the genetic causes of children with unexplained ID and determined the clinical characteristics of children with genetic confirmation.

## Method

### Study participants and design

This cohort study focused on children with unexplained ID who visited the child development and medical genetics clinic of a tertiary medical center between September 2020 and February 2023 for unexplained ID and underwent genetic testing. Inclusion criteria were (1) children with clinical diagnosis of ID, (2) absence of prenatal or perinatal problems, exogenous factors such as alcohol or drug ingestion or infections during pregnancy, or events that could cause ID, (3) found to have no abnormalities in metabolic testing (e.g., newborn metabolic screening, thyroid function test, plasma amino acid, plasma acylcarnitine, and urine organic acid), and (4) in cases of suspected diseases, disease-specific genetic tests were conducted (eg., triplet repeat primed PCR to exclude myotonic dystrophy or fragile X syndrome, and the methylation of *SNRPN* was analyzed via methylation-specific PCR restriction fragment length polymorphism to exclude Prader–Willi and Angelman syndromes), and no specific abnormalities were detected.

**Figure 1:**
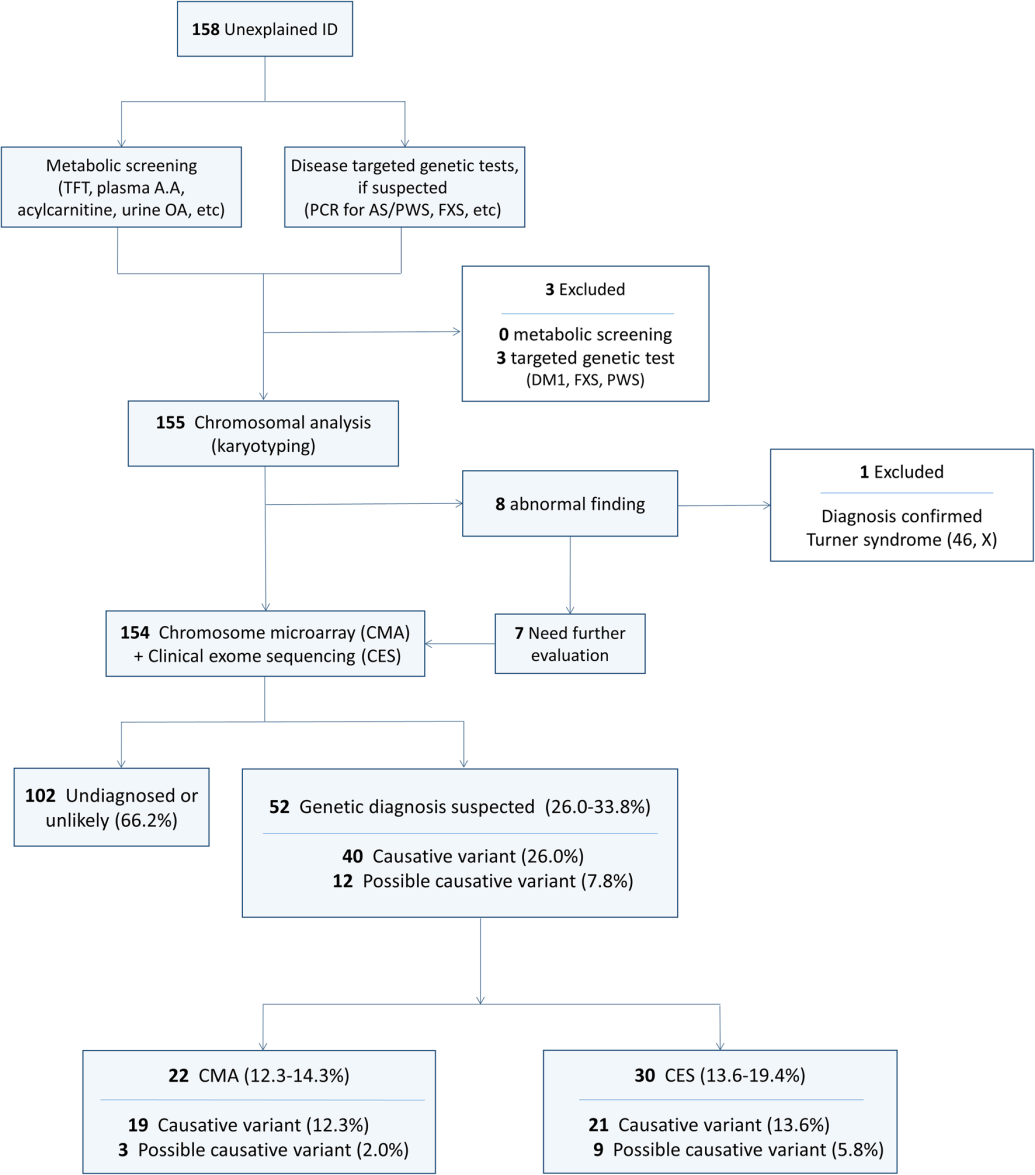
Flowchart of the study. ID, intellectual disability; TFT, thyroid function test; A.A, amino acid; OA, organic acid; PCR, polymerase chain reaction; AS, Angelman syndrome; PWS, Prader-Willi syndrome; FXS, Fragile X syndrome; DM1, myotonic dystrophy type 1.

In this study, if no significant abnormalities were detected in the initial evaluation, a 550-band level chromosomal analysis of peripheral blood lymphocytes was performed. If chromosomal abnormalities were detected and further testing was unnecessary, the children were excluded from the CMA and CES studies. However, if abnormalities were found but required CMA testing for accurate diagnosis and if no abnormality was found, the children were included in the study and underwent simultaneous CMA and CES tests. Figure 1 shows the flow chart of the study. Two clinicians determined the eligibility of the study participants based on clinical examination, developmental assessment, and diagnostic tests.

DSM-5 criteria were used to select children, and those with an IQ score of < 70 measured by the Wechsler Intelligence Scale for Children-V (WISC-V) or Wechsler Primary and Preschool Scale Intelligence-IV (WPPSI-IV) were included as study subjects. Children who could not perform the Wechsler intelligence test due to poor understanding of the test or whose test results were unreliable were included if they were suitable for ID based on clinical findings from the Bayley Scales of Infant Development, Beery Test of Visual-Motor Integration, Vineland Social Maturity scale, and Pediatric Evaluation of Disability Inventory tests. Children who were tested before 36 months of age were included as study subjects following re-evaluation at 36 months of corrected age.

Also, all children included in this study underwent brain MRI to confirm that ID was not caused by brain lesions, such as brain injury, periventricular leukomalacia, hypoxic ischemic encephalopathy, or encephalitis, and to identify the structural abnormality of the brain. In instances when sedation failed or parental consent was not obtained, brain MRI was not conducted. Children who were found to have no abnormality in the above tests and did not have any other suspected recognizable syndromes were included in this study and underwent further genetic testing.

Only children whose parents consented to genetic testing were included, and informed consent from the patient's guardian was collected. The Institutional Review Board of Incheon St. Mary’s hospital approved that this study was performed in accordance with relevant guidelines and regulations (ethics approval number: OC22RISI0155).

### Clinical manifestation

We investigated other ID-associated phenotypes in children. (1) Gross motor delay—presence of general hypotonia or inability to gait independently after 18 months of age, (2) Epilepsy—two or more clinically distinct seizure events accompanied by electroencephalography (EEG) abnormalities, (3) Facial dysmorphism—according to anatomical facial structures^15^, (4) Congenital structural anomalies—external or internal major and minor structural anomalies, (5) Microcephaly (head circumference less than the 3rd percentile) or macrocephaly (head circumference above the 97th percentile).

### Molecular genetic diagnosis

#### CMA and CES tests

CMA and CES tests were initially performed on the proband alone. Subsequently, parent testing was performed based on the obtained results. CMA and Sanger sequencing were performed for CNV detection and sequence variant analysis, respectively.

#### CMA and targeted gene panel CES tests

Genomic DNA was extracted from leukocytes from peripheral blood. CMA was performed using CytoScan Optima chips and Cytoscan^®^ 750K (Affymetrix, Santa Clara, CA, USA). Data analysis was performed using Chromosome Analysis Suite 3.1 software with human genome build hg19.

For CES testing, a TruSight One-Expanded panel was used. Genes were additionally targeted and tested according to the patients' coexisting phenotypes (e.g., epilepsy and structural anomaly). Libraries were prepared with the Illumina™ Truseq DNA kit, and sequencing was performed using the Illumina™ Nextseq platform. SNVs were verified using Sanger sequencing.

#### Variants interpretation

CMA-identified CNVs were classified as pathogenic, likely pathogenic, variants of uncertain significance (VUS), likely benign, and benign according to ACMG and Clinical Genome Resource (ClinGen) guidelines (2020) of semiquantitative point-based scoring in consideration of size and genomic contents of CNV, dosage sensitivity predictions and inheritance pattern; moreover, databases and published literature such as DGV (database of genomic variants), ClinGen (clinical genome resource), OMIM, GeneReviews, DECIPHER, and UCSC were also considered^16,17^. Also, we utilized in-house CMA database (containing over 5000 cases) for CNVs interpretation. CES-identified sequence variants were also classified into pathogenic, likely pathogenic, VUS, likely benign, and benign according to the ACMG guidelines^18^. Parental tests were performed to determine inheritance. Considering the clinical manifestations and other test results, parent tests, inheritance pattern, pedigree, and previous literature reports, as well as the classification of each CNV and sequence variant, the significance of variant was finally determined as a (1) causative variant—having sufficient evidence, (2) a possible causative variant—classified as a likely pathogenic variant according to the ACMG classification and clinically relevant, but parental test was not performed. The variants were determined by a clinical laboratory geneticist and two clinicians.

### Statistical analysis

Statistical analysis was performed using SPSS version 28.0 for Windows (IBM Corp., Armonk, NY). Pearson chi square and Fisher’s exact tests were used to compare the causal and possible causative variant detection rates between the presence of the coexisting phenotype. The diagnostic yields of CMA and CES were compared using McNemar's test. A *P*-value < 0.0083 was considered statistically significant based on the Bonferroni method.

## Results

A total of 154 children (110 male children [71%]; 44 female children [29%]) were included. Table 1 presents the clinical characteristics of the children. The mean age at which genetic testing was performed was 51.9 months. Of the included children, 59 (38.3%) had delayed gross motor development, 8 (5.2%) had epilepsy, 49 (31.8%) had facial dysmorphism, 33 (21.4%) had congenital structural anomaly, and 28 (18.8%) showed microcephaly (17 children) or macrocephaly (11 children). Brain MRI performed on 149 children revealed abnormalities in 17 children (11.4%). Congenital structural anomalies included congenital heart defects (ventricular and atrial septal defects, ascending aorta dilatation, and pulmonary atresia), craniosynostosis, microtia with external auditory canal agenesis, microphthalmia, general joint laxity, multiple joint contractures, syndactyly, and osteogenesis imperfecta.

**Table 1:** Demographic characteristics of subjects.

Characteristics	No. (%)
Child sex	
Male	110 (71.4)
Female	44 (28.6)
Child age, mean (SD), mo	51.9 (23.1)
Coexisted phenotypes	
Gross motor delay	59 (38.3)
Epilepsy	8 (5.2)
Facial dysmorphism	49 (31.8)
Congenital structural anomaly	33 (21.4)
Abnormal head circumference	28 (18.2)
Microcephaly	17 (11.0)
Macrocephaly	11 (7.1)
Brain MRI abnormalities (*N* = 149)	17 (11.4)

### Analysis of variants

Combining both CMA and CES tests, the causative variants were found in 26.0% (40/154), whereas the possible causative variants were found in 7.8% (12/154). The overall diagnostic yield was found to be 26.0–33.8% (Table 2). CMA and CES tests showed no statistically significant difference in the genetic diagnostic rate. Detailed descriptions of the variants found are provided in the Supplementary material (Supplemental Tables S1, 2).

**Table 2:** Results of chromosomal microarray analysis and clinical exome sequencing (*n* = 154).

	No. of children according to methods (%)		
	CMA	CES	Total
Causative variant	19 (12.3)	21 (13.6)	40 (26.0)
Possible causative variant	3 (2.0)	9 (5.8)	12 (7.8)

CMA, chromosomal microarray analysis; CES, clinical exome sequencing.

CMA revealed causative variants in 19 children (12.3%) and possible causative variants in 3 children (2.0%). Of the 19 children with identified causative variants in the CMA test, 7 (36.8%) underwent parental testing; three children who were diagnosed with a possible causative variant did not undergo parental testing. The causative CNVs identified were 14 deletions and 5 duplications.

CES testing revealed causative variants in 21 children (13.6%) and possible causative variants in 9 children (5.8%). Among the children with identified causative variants, 14 (66.7%) cases underwent parental test. Among them, 12 cases were found to be de novo; one was inherited in an autosomal recessive (AR) manner, and one was inherited in an autosomal dominant (AD) manner and had a similar ID phenotype as her mother. Out of 22 variants, 12 were novel. Of the 21 cases, 19 showed autosomal dominant (AD) inheritance, and the remaining 2 cases had AR and X-linked recessive inheritance. Of the 9 children with possible causative variants found, 1 (11.1%) had performed parent testing.

### Association with clinical phenotypes

The analysis of the relationship between coexisting clinical features and diagnostic yield revealed that the possibility of genetic diagnosis was significantly higher when gross motor delay (odd ratio (OR) 6.69; 95% confidence interval (CI) 3.20–14.00; P < 0.001), facial dysmorphism (OR 9.34; 95% CI 4.29–20.30; P < 0.001), congenital structural anomalies (OR 3.62; 95% CI 1.63–8.04; P = 0.001), and microcephaly or macrocephaly (OR 4.87; 95% CI 2.05–11.60; P < 0.001) were coexisting. The diagnostic yield did not increase significantly in individuals with epilepsy or aberrant brain MRI, and no significant difference existed between microcephaly and macrocephaly (Table 3). In patients with verified genetic diseases, no significant difference in clinical features was observed between the CMA and CES methods.

**Table 3:** Coexisting phenotypes with or without causative and possible causative variants.

Coexisting phenotypes	Identification of causative or possible causative variants			
	Yes (*N* = 52)	No (*N* = 102)	OR (95% CI)	*P*-value
Gross motor delay	35	24	6.69 (3.20–14.00)	< 0.001
Epilepsy^a^	2	6	0.64 (0.13–3.29)	0.718
Facial dysmorphism	33	16	9.34 (4.29–20.30)	< 0.001
Congenial structural anomaly	19	14	3.62 (1.63–8.04)	0.001
Micro/macrocephaly	18	10	4.87 (2.05–11.60)	< 0.001
Brain MRI abnormality	6	11	1.06 (0.37–3.04)	0.922

OR, odds ratio; CI, confidence interval.

^a^Fisher’s exact test.

A total of 63 children (40.9%) had only ID without other phenotypes, causative variants were identified in 4 children, and possible causative variants were found in 3 children. The diagnostic rate among children with only ID was 6.3%–11.1%, whereas that among children with other accompanying phenotypes was 39.6%–49.5%.

### Impact of chromosomal analysis

Seven cases were observed with abnormalities on chromosomal analysis, with one having balanced reciprocal translocation (t(8;9)(q24.3;q33)). The proband CMA revealed 3p12.1q13.11(84,008,316–103,098,838) LOH (19.1 Mb) and 10p11.21(34,928,832–35,334,487) × 3 (405 kb), without detecting balanced translocation. In the other six patients, the abnormalities revealed by chromosomal analysis were also found in CMA, providing more detailed information.

## Discussion

We investigated the diagnostic yield of CMA and CES in children with ID who were excluded from brain injury, metabolic problems, and well-known syndromic diseases, such as fragile X, Prader–Willi, and Angelman syndromes. The analysis results of the accompanying clinical phenotypes of children diagnosed with genetic disease indicated an overall diagnostic yield of 26.0–33.8%, with CMA contributing 12.3–14.3% and CES contributing 13.6–19.4%. However, the two tests showed no significant difference in diagnostic yield. A coexistence of gross motor delay, facial dysmorphism, congenital structural anomaly, and microcephaly or macrocephaly can significantly increase the probability of a genetic diagnosis by CMA and CES. These phenotypes were also unrelated to the diagnostic yields of CMA and CES.

ES/GS has recently been recognized as a first- or second-tier test for children with ID^7^. Despite its potential, cost and time-effectiveness remain significant obstacles impeding its widespread application. The fact remains that not all regions can conduct high-quality GS due to technological limitations, lack of experts, and resource constraints, highlighting the need to establish clinical guidelines suitable for the current clinical setting. In addition, less than 5% of cases gained further genetic diagnosis through GS after receiving negative ES/CMA results^7,9,14,19,20^. Given the current level of technology and cost, the universal application of GS in all cases may be challenging^21^.

Notably, our study had a lower diagnostic yield than previous studies, which reported 14.7–36% for CMA and 29–55.7% for ES in children with ID^6,10,12,22–27^. This difference may be due to our inclusion of children with ID without other phenotypes provided they met the inclusion criteria and inclusion of children regardless of their disability severity. In fact, a severe degree of ID is associated with a higher genetic diagnostic rate; for instance, a study of patients with ID having an IQ of < 50 reported a diagnostic yield of 55.7% for ES^6,27^.

In this study, most causative variants diagnosed with CES showed AD inheritance (87.0%, 19/21). The AR case was Cohen syndrome. Biallelic pathogenic variants from the mother and father caused Cohen syndrome due to biallelic *VPS13B* pathogenic variants *in trans* configuration (c.5809_5810del and exon 32 deletion). XLR disease was detected in a child diagnosed with Duchenne muscular dystrophy (DMD) caused by a pathogenic variant in DMD (NM_004006.3:c.9563+1G>A, intron 65). This variant was classified as pathogenic according to the ACMG guidelines, and the patient was diagnosed as a causal variant because of his phenotype—hypotonia and gross motor delay. However, as the proband showed ID with a measured Wechsler test IQ of 41 and microcephaly, it was difficult to determine if ID was also caused by a pathogenic variant of DMD. The possibility of the presence of an additional pathogenic variant could not be ruled out. Nevertheless, ID is a well-known DMD comorbidity, and approximately 30% of patients with DMD have ID with an IQ of 70^28,29^. Notably, the child belonged to the dystrophin Dp71 group, and the Dp71 group revealed to have a significantly lower IQ compared to other groups (mean IQ score 46)^30^. It is associated with disruption of dystrophin isoforms. Also, there are previous reports of DMD patients with microcephaly^31^. Nonetheless, there may be limitations in interpreting the results because we cannot rule out the possibility of the coexistence of other variants.

The de novo pathogenic variants were the major cause of ID in this study (85.7%, 12/14), as in previous reports^3,32,33^. The most common diseases were Rubinstein-Taybi syndrome (*CREBBP* c.3292del, *CREBBP* c.6587_6588del, *EP300* c.5961_5962del), Sotos syndrome (*NSD1* c.2645C>G, *NSD1* c.1492C>T (2 probands, monozygotic twin)), and *DNMT3A*-related disorders (*DNMT3A* c.1258A>T, *DNMT3A* c.1279G>T, and *DNMT3A* c.1012_1014+3del) in 3 probands, respectively.

Our analysis of the genotype–phenotype correlation and comparison of the diagnostic rate between CMA and CES for each phenotype did not reveal a significant difference between the two tests. Also, the identified variants varied among patients with the same phenotype, and all different causative variants were observed in patients with isolated ID. Therefore, in most cases other than well-known syndromic ID, phenotypes alone cannot predict genetic abnormalities in advance and do not provide information on whether the cause lies with SV or sequence variant.

A significantly increased diagnostic yield was observed in children with gross motor delay, facial dysmorphism, congenital structural anomaly, and microcephaly or macrocephaly, with epilepsy unrelated to diagnostic rate, corroborating previous studies^6,34,35^. This could guide clinicians to recommend more aggressive testing for caregivers of children with certain phenotypic features or provide a rationale for more active surveillance for the presence of certain phenotypes in children with isolated ID, although ID-associated phenotypes warrant further research.

The current study did not find any cases where chromosomal analysis provided additional diagnostic benefits, so the scant diagnostic value of chromosomal analysis in cases of ID was highlighted. Only one case was identified with Turner Syndrome (46, X), where CMA/CES tests were not conducted due to the confirmation of the disease through chromosome analysis. In fact, balanced chromosomal translocation and inversion may not be diagnosed through CMA alone, and chromosome analysis may be required in such cases. Previous studies revealed that balanced translocation accounted for 1%–2% of ID cases and that in cases where CMA was normal, only 1.08% showed abnormalities on chromosomal analysis^36–39^.

## Limitations

Possible limitations of this study include the exclusion of some children with ID due to the cost of diagnostic tests, despite objective and identical inclusion or exclusion criteria. While we performed singleton CES of affected probands, performing trios testing may have increased the diagnostic yield. Nevertheless, we attempted to improve the study's quality by conducting parental tests of identified variants and promoting in-depth communication between clinicians and geneticists. Notably, epilepsy is often diagnosed in later childhood, which could lead to a hasty diagnosis of epilepsy-free during genetic testing. However, the presence of epilepsy may not be significant for genetic testing when diagnosing ID. Finally, variable penetrance, expressivity, and phenotypic variation may have resulted in some cases being unclearly diagnosed.

## Conclusions

This study demonstrated that the overall diagnostic yield of concurrent CMA and CES was 26.0–33.8%, with CMA contributing 12.3–14.3% and CES contributing 13.6–19.4% for children with unexplained ID. Coexistence of certain phenotypes such as gross motor delay, facial dysmorphism, congenital structural anomaly, and microcephaly or macrocephaly further increased the diagnostic rate. Overall, this study suggests that the concurrent application of CMA and CES tests is an effective approach for enhancing the genetic diagnosis in children with unexplained ID.

### Supplementary Information

Supplementary Tables.

## Data Availability

The datasets used and/or analyzed during the current study available from the corresponding author on reasonable request and data of the variants are publicly available from ClinVar database: ClinVar, [https://www.ncbi.nlm.nih.gov/clinvar/submitters/506870/], accession number: SCV004023327, SCV004024471, SCV004023329 to SCV004023332, SCV004023334, SCV004023335, SCV004023337 to SCV004023340, SCV004023342 to SCV004023347, SCV000882768, SCV004023311 to SCV004023325, SCV003845958, SCV004023407, SCV004024266, SCV004023326, SUB14039259, SUB14039235, SUB14045015.
