# Supplementary material for: Combining chromosomal microarray and clinical exome sequencing for genetic diagnosis of intellectual disability
Source: Sci Rep. 2023 Dec 20;13:22807. doi: 10.1038/s41598-023-50285-z (PMC10739828; doi:10.1038/s41598-023-50285-z)
Supplement: Supplementary file 1 — Supplementary Tables. [file 41598_2023_50285_MOESM1_ESM.docx]

**Supplementary Table S1. Clinical Phenotypes and Causative Variants Confirmed with Chromosomal Microarray Analysis (*N* = 19)**

| Patient number | Diagnosis age | Sex | Molecular diagnosis | Classification* (score) | Major OMIM genes | Clinical features combined with intellectual disability | ClinVar accession number |
| --- | --- | --- | --- | --- | --- | --- | --- |
| 1 | 3 y | M | 18q22.1q23 (66,057,406-78,014,123)  x 1 [12.0 Mb] | P  (2.2) | *DOK6, CD226, RTTN, SOCS6, CBLN2, NETO1, FBXO15, CYB5A, FAM69C, CDNP2, CNDP1, ZNF407, TSHZ1, ZNF236, ZNF516, MBP, GALR1, TIMM21* | gross motor delay, facial dysmorphism, bifid uvula, mitral and tricuspid valve regurgitation, ascending aorta dilatation | SCV004023327 |
| 2 | 4 y | F | 7q11.23 (72,718,277-74,142,256) x 3  [1.4 Mb] | P  (1.6) | *NSUN5, TRIM50, FKBP6, FZD9, BAZ1B, BCL7B, TBL2, MLXIPL, VPS37D, WBSCR22, STX1A* | gross motor delay, facial dysmorphism, small ear, high arch palate, ascending aorta dilatation | SCV004024471 |
| 3 | 5 y | M | 8q24.13q24.21 (124,534,271-129,054,138) x 4~5 [4.5 Mb] †  8q24.21q24.3 (131,138,343-143,473,913) x 3 [12.3 Mb] † | P  (2.05)  P  (2.5) | *FBXO32, ANXA13, RNF139, NDUF89, NTSS1, SQLE, KIAA0196, TRIB1, FAM84B, FRNCR1, POU5F1B, MYC, PVT1, ASAP1, ADCY8, EFR3A, OC90, HHLA1* | gross motor delay, atrial septal defect | SUB14045015  SCV004023329 |
| 4 | 6 y | M | 17q11.2q12 (30,572,862-35,843,988)  x 1 [5.3 Mb] † | P  (2.2) | *ZNF207, PSMD11, CDK5R1, MYO1D, SPACA3, ASIC2, CCL2, CCL7, PEX12, AP2B1, RASL10B* | gross motor delay, speech delay, joint hypermobility, undescended testis | SCV004023330 |
| 5 | 15 mo | M | 10q25.1q26.3 (111,378,692-135,427,143) x 3 [24.0 Mb] † | P  (3.1) | *XPNPEP1, ADD3, MXI1, SMNDC1, DUSP5, SMC3, RBM20, PDCD4, BBIP1, SHOC2, ADRA2A, GPAM, TECTB, ACSL5, VTI1A, TCF7L2* | gross motor delay, facial dysmorphism | SCV004023331 |
| 6 | 13 mo | M | 2p25.3 (12,770-2,832,894) x 1 [2.8 Mb]  4p16.3p15.1 (68,345-34,512,694) x 3  [34.4 Mb] | P  (1.1)  P  (2.9) | *FAM110C, ACP1, TMEM18, SNTG2, TPO, PXDN, MYT1L, ZNF141, PDE6B, ATP5I, MYL5, CPLX1* | gross motor delay, epilepsy, facial dysmorphism, atrial septal defect, strabismus, overlapped toes | SUB14039235  SCV004023332 |
| 7 | 6 y | M | 7q11.23(72,664,461-74,162,586) x 3  [1.2 Mb] [P] | P  (1.85) | *NSUN5, TRIM50, FKBP6, FZD9, BAZ1B, BCL7B, TBL2, MLXIPL, VPS37D, MLXIPL, VPS37D, WBSCR22, STX1A, CLDN3, CLDN4* | (-) | SCV004023334 |
| 8 | 35 mo | M | 10q26.13q26.3 (123,477,898-135,427,143) x 1 [11.9 Mb] | P  (2.2) | *ATE1, NSMCE4A, TACC2, PLEKHA1, ARMS2, HTRA1, DMBT1, PSTK, IKZF5, ACADSB, HMX3, HMX2, BUB3, GPR26, CHST15, OAT, FAM175B* | gross motor delay, facial dysmorphism, craniosynotosis, ventricular septal defect, strabismus | SCV004023335 |
| 9 | 4 y | F | 15q11.2 (22,770,421-23,282,799) x 1  [512 Kb] | P  (1.1) | *TUBGCP5, CYFIP1, NIPA2, NIPA1* | (-) | SCV004023337 |
| 10 | 3 y | M | 1p36.33p36.31 (1,957,148-6,553,454) x 1  [4.6 Mb] † | P  (2.2) | *GABRD, PRKCS, C1orf86, SKI, PEX10, PLCH2, PANK4, HESS, TNFRSF14, ACTRT2, PRDM16, MEGF6, MIR551A, TPRG1L, WRAP73, TP73* | facial dysmorphism, chiari malformation, corpus callosum dysplasia | SCV004023338 |
| 11 | 3 y | M | 6q21q22.31 (112,713,854-124,105,184) x 1  [11.4 Mb] | P  (2.2) | *MARCKS, HDAC2, HS3ST5, FRK, COL10A1, TSPYL1, DSE, TRAPPC3L, RSPH4A, KPNA5, GPRC6A, RFX6, VGLL2, ROS1, GOPC, NUS1* | gross motor delay, facial dysmorphism, microcephaly, polysyndactyly | SCV004023339 |
| 12 | 21 mo | F | 15q24.1q24.2 (72,943,184-76,085,232) x 1  [3.1 Mb] † | P  (2.0) | *GGS4, ADPGK, NEO1, HCN4, NPTN, CD276, LOXL1, STOML1, PML, GOLGA6A, ISLR2, ISLR* | gross motor delay | SCV004023340 |
| 13 | 35 mo | F | 10q26.2q26.3 (128,289,206-135,427,143) x 1  [7.1 Mb] | P  (2.0) | *DOCK1, NPS, PTPRE, MKI67, MGMT, EBF3, GLRX3, PPP2R2D, BNIP3, JAKMIP3, DPYSL4, INPP5A, GPR123, UTF1, VENTX, ADAM8* | gross motor delay, atrial septal defect | SUB14039259 |
| 14 | 4 y | F | 15q13.2q13.3 (31,073,735-32,446,830) x 1  [1.4 Mb] | P  (1.3) | *FAN1, TRPM1, MIR211, KLF13, OTUD7A, CHRNA7* | facial dysmorphism, ascending aorta dilatation | SCV004023342 |
| 15 | 3 y | F | 15q11.2q13.2 (22,770,421-30,386,398) x 4  [7.6 Mb] | P  (2.9) | *TUBGCP5, CYFIP1, NIPA2, NIPA1, MKRN3, MAGEL2, NDN, PWRN2, PWRN1, NPAP1, SNRPN, PWAR5, SNORD116-1, IPW, PWAR1, UBE3A* | gross motor delay, facial dysmorphism | SCV004023343 |
| 16 | 5 y | M | 22q11.21 (18,916,842-20,311,810) x 3  [1.4 Mb] | P  (2.9) | *PRODH, DGCR2, DGCR14, TSSK2, SLC25A1, GSC2, CLTCL1, HIRA, MRPL40, UFD1L, CDC45, CLDN5, GP1BB, TBX1, GNB1L, TXNRD2, COMT* | facial dysmorphism, ascending aorta dilatation | SCV004023344 |
| 17 | 28 mo | F | 11p15.5 (268,586-748,873) x 1  [480 Kb]† | LP  (0.9) | *NLRP6, IFITM5, IFITM2, IFITM1, IFITM4, PKP3, SIGIRR, PTDSS2, RNH1, HRAS, RASSF7, MIR210, IRF7, CDHR5, SCT, DRD4, DEAF1, EPS8L2* | gross motor delay, facial dysmorphism, microcephaly | SCV004023345 |
| 18 | 6 y | M | 1q21.1 (145,382,123-145,792,051) x 1  [410 Kb]† | LP  (0.9) | *NBPF20, NBPF10, HFE2, TXNIP, POLR3GL, ANKRD34A, RBM8A, GNRHR2, PEX11B, ITGA10* | (-) | SCV004023346 |
| 19 | 4 y | M | 15q11.2 (22,770,421-23,195,725) x 1  [425 Kb] | P  (1.1) | *TUBGCP5, CYFIP1, NIPA2, NIPA1, LOC283683, WHAMMP3* | (-) | SCV004023347 |

*This indicates the classification of variants using ACMG-ClinGen guidelines (2020). The copy-number variants classified as pathogenic have a score of 0.99 or more points, and those classified as likely pathogenic have scores ranging from 0.90 to 0.98. P, pathogenic; LP, likely pathogenic. †Parental testing was performed, confirming that the variant occurred de novo. **Supplementary Table S2. Clinical Phenotypes and Causative Variants Confirmed with Clinical Exome Sequencing (*N* = 21)**

| Patient number | Diagnosis age | Sex | Diagnosis | Molecular diagnosis | Classification*  (category) | Clinical features combined with intellectual disability | ClinVar accession number |
| --- | --- | --- | --- | --- | --- | --- | --- |
| 1 | 5 y | M | Duchenne muscular dystrophy | *DMD* (NM_004006.3):c.9563+1G>A | P  (PVS1+PS1+PM2  +PP4+PP5) | gross motor delay | SCV000882768 |
| 2 | 3 y | F | Cohen syndrome | *VPS13B* (NM_152564.5):c.5734_5735del  (p.Ile1912CysfsTer11) [from mother]  *VPS12B* exon 32 deletion on MLPA [from father] | P  (PVS1+PM2+PP4+PP5) | gross motor delay, facial dysmorphism, microcephaly, myopia, ventricular septal defect | SCV004023311 |
| 3 | 9 y | M | Cantú Syndrome | *ABCC9* (NM_020297.4):c.3346C>T†  (p.Arg1116Cys) | P  (PS1+PM1+PM2+PM5  +PP3+PP4+PP5) | facial dysmorphism, aortic valve stenosis, hippocampus hypoplasia | SCV004023312 |
| 4 | 20 mo | M | Developmental and epileptic encephalopathy 69 | *CACNA1E* (NM_000721.4):c.6529C>T†  (p.Gln2177Ter) | P  (PVS1+PS2+PM1  +PM2+PP3+PP4) | gross motor delay, facial dysmorphism | SCV004023313 |
| 5 | 31 mo | F | Snijders Blok-Campeau syndrome | *CHD3* (NM_001005273.3):c.3431A>T‡ (p.Asn1144Ile) | LP  (PM1+PM2+PP1  +PP3+PP4) | gross motor delay, facial dysmorphism | SCV004023314 |
| 6 | 3 y | M | Rubinstein-Taybi Syndrome | *CREBBP* (NM_004380.3):c.3292del  (p.Leu1098Ter) | P  (PVS1+PS1+PM2  +PM4+PP4+PP5) | gross motor delay, facial dysmorphism, strabismus | SCV004023315 |
| 7 | 4 y | M | Rubinstein-Taybi Syndrome | *EP300* (NM_001429.4):c.5961_5962del (p.Gly1988AspfsTer84) | P  (PVS1+PM2+PM6+PP4) | facial dysmorphism, microcephaly, hearing loss, hyperopia, multiple unidentified bright objects in white matter | SCV004023316 |
| 8 | 6 y | M | Rubinstein-Taybi Syndrome | *CREBBP*(NM_004380.3):c.6587_6588dup† (p.Leu2197SerfsTer106) | P  (PVS1+PS2+PM2+PP4) | gross motor delay, facial dysmorphism, broad thumbs, skew foot, short stature | SCV004023317 |
| 9 | 3 y | M | Cowden Syndrome | *PTEN* (NM_001304718.2):c.203T>C  (p.Leu68Pro) | LP  (PM1+PM2+PP4+PP5) | gross motor delay, facial dysmorphism, large arachnoid cyst, macrocephaly | SCV004023318 |
| 10 | 5 y | M | Cowden syndrome | *PTEN* (NM_000314.8):c.697C>T†  (p.Arg233Ter) | P  (PVS1+PS1+PS2+PM2  +PM4+PP4+PP5) | macrocephaly | SCV004023319 |
| 11 | 27 mo | M | Bainbridge-Ropers syndrome | *ASXL3* (NM_030632.3):c.1628_1629del† (p.Leu543HisfsTer12) | P  (PVS1+PS1+PS2+PM2  +PM4+PP4+PP5) | gross motor delay, facial dysmorphism, fingers arthrogryposis | SCV004023320 |
| 12 | 32 mo | M | CHARGE syndrome | *CHD7* (NM_017780.4):c.5405-7G>A† | P  (PS1+PS2+PM2  +PP4+PP5) | gross motor delay, facial dysmorphism, cryptorchidism, hearing loss | SCV004023321 |
| 13 | 10 mo | M | SBBYSS syndrome | *KAT6B* (NM_012330.4):c.4911_4921del (p.Val1638AlafsTer27) | P  (PVS1+PS1+PM2 +PM4+PP4+PP5) | gross motor delay, facial dysmorphism, ptosis, microcephaly, atrial septal defect | SCV004023322 |
| 14 | 19 mo | F | Impaired intellectual development and distinctive facial features with or without cardiac defects | *MED13L* (NM_015335.5):c.5005G>A  (p.Asp1669Asn) | LP  (PS2+PM2+PP4+PP5) | gross motor delay, facial dysmorphism, strabismus | SCV004023323 |
| 15 | 4 y | M | Sotos syndrome | *NSD1* (NM_022455.5):c.2645C>G  (p.Ser882Ter) | P  (PVS1+PS1+PM1+PM2  +PM4+PP4+PP5) | gross motor delay, facial dysmorphism, craniosynostosis | SCV004023324 |
| 16,17 | 4 y | M | Sotos syndrome | *NSD1*(NM_022455.5):c.1492C>T†  (p.Arg498Ter) | P  (PVS1+PS1+PS2+PM2  +PM4+PP4+PP5) | gross motor delay, facial dysmorphism, macrocephaly | SCV004023325 |
| 18 | 5 y | F | Heyn-Sproul-Jackson syndrome | *DNMT3A*(NM_175629.2):c.1012_1014+3del† | P  (PVS1+PS1+PS2+PM2  +PP4+PP5) | gross motor delay, facial dysmorphism, craniosynostosis, microcephaly | SCV003845958 |
| 19 | 9 y | F | Tatton-Brown-Rahman Syndrome | *DNMT3A* (NM_175629.2):c.1258A>T  (p.Lys420Ter) | P  (PVS1+PS1+PS2+PM2  +PP4+PP5) | gross motor delay, facial dysmorphism, macrocephaly | SCV004023407 |
| 20 | 5 y | M | Tatton-Brown-Rahman Syndrome | *DNMT3A* (NM_175629.2):c.1279G>T†  (p.Glu427Ter) | P  (PVS1+PS2+PM2  +PP4+PP5) | gross motor delay, facial dysmorphism | SCV004024266 |
| 21 | 3 y | F | Intellectual developmental disorder with autism and macrocephaly | *CHD8* (NM_001170629.2):c.3623G>T† (p.Cys1208Phe) | P  (PS2+PM1+PM2  +PP3+PP4+PP5) | gross motor delay, facial dysmorphism, macrocephaly | SCV004023326 |

The identified variants are all heterozygous. *This indicates the classification of variants using ACMG-AMP guidelines (2015). P, pathogenic; LP, likely pathogenic; PVS, pathogenic very strong; PS, pathogenic strong; PM, pathogenic moderate; PP, pathogenic supporting. †Parental testing was performed, confirming that the variant occurred de novo. ‡ same variant and phenotype as her mother.
